# Design and Antischizophrenic Studies of 1,3-disubstituted Chalcone Derivatives: *In-silico* Molecular Docking Approach

**DOI:** 10.21315/tlsr2025.36.3.10

**Published:** 2025-10-31

**Authors:** Abdulqadir Okhayole Zubair, Suleiman Danladi, Umar Idris Ibrahim, Mahdiyu Kogi Jibril, Salim Ilyasu

**Affiliations:** 1 Department of Pharmaceutical and Medicinal Chemistry, Faculty of Pharmaceutical Sciences, Bayero University, Kano, Kano State, Nigeria; 2 Department of Clinical Pharmacy and Pharmacy Practice, Faculty of Pharmacy, Universiti Sultan Zainal Abidin, 22200 Besut, Terengganu, Malaysia; 3 Department of Pharmaceutics and Pharmaceutical Technology, Faculty of Pharmaceutical Sciences, Bayero University, Kano, Kano State, Nigeria

**Keywords:** Design, Antischizophrenic, Chalcone, *In silico*, Molecular-docking

## Abstract

Schizophrenia is a psychiatric disorder that affects a person’s ability to think, feel and behave clearly. The pathophysiology of the disease stems from the overexpression of dopamine neurotransmission and deficiency of glutamate activity at glutamate synapse in the brain. Considering the significant global burden of the disease, lack of complete efficacy using the current medications and variety of adverse effects associated with their use, and the huge opportunity created by Computer-Aided Drug Design, it is therefore important and possible to come up with drug leads that will have improved efficacy and reduced side effects. This research is thus aimed to design, evaluate the pharmacokinetic properties, and the in silico antischizophrenic activity of novel 1,3-disubstituted chalcones. Ten compounds were designed using ChemDraw Ultra 7.0 using similarity approach and their oral bioavailability was predicted using SwissADME, toxicity was predicted using PROTOX 3.0 and docking studies were carried out using AutoDock Vina through Chimera 1.11.2. The 10 designed compounds were predicted to have excellent oral bioavailability and lead-likeness properties, easy synthesisability and a relatively safer toxicity profile than the reference compound clozapine. The compounds were evaluated to have higher docking scores between −8.3 to −9.5 compared to clozapine with a docking score of −8.8 when docked against dopaminergic D2 receptor. In addition, the compounds have close binding scores (−6.0 to −6.6) compared to clozapine (−6.7) when docked against N-Methyl-D-aspartic Acid (NMDA) receptor, suggesting their use as potential antischizophrenic agents.

**HIGHLIGHTS**

- Ten novel 1,3-disubstituted chalcones were designed as potential antischizophrenic agents.
- All compounds showed excellent drug-likeness, bioavailability and CNS penetration.
- Compounds C6, C7 and C8 demonstrated higher binding for the dopamine D2 receptor than the standard drug clozapine, while all compounds and clozapine showed comparable affinity for the glutamatergic N2D receptor, indicating their promising antischizophrenic activity.

## INTRODUCTION

Schizophrenia is a challenging, common and complex psychiatric disorder affecting about 0.3% of the population globally (World Health Organization [WHO] 2022) comprising three major groups of symptoms: positive, negative and cognitive symptoms (Li *et al*. 2016). It has been linked to the hypofunction of the glutamate NMDA receptors in the prefrontal cortex of the brain, hyperfunction of the striatal dopaminergic D2 receptors and others, including serotonergic and cholinergic receptors (Li *et al*. 2016; Stahl 2018; Paul *et al*. 2022), all contributing to the development of its different symptoms (Carbon & Correll 2014).

The current therapy methods for the management of schizophrenia involve the use of different classes of drugs including the typical antipsychotics which were first discovered in the 1950s such as chlorpromazine, thioridazine, haloperidol, etc., which all act via blockade of the dopaminergic D2 receptors in the brain thereby making them very effective in the alleviation of positive symptoms of schizophrenia such as delusions (Snyder *et al*. 1974). Patients on this class of drugs often experience various side effects, including extrapyramidal side effects such as tremors, involuntary movements, stiffness, etc. (Blair & Dauner 1992), tardive dyskinesia, galactorrhea and gynecomastia and others (Li *et al*. 2016). Moreover, this class of drugs do not affect the negative and cognitive symptoms of schizophrenia (Carbon & Correll 2014).

Another class of drugs widely used in schizophrenia management is the atypical antipsychotics, such as clozapine, risperidone, olanzapine, etc., which were designed to mitigate the extrapyramidal side effects seen with the use of the typical class by targeting the serotonin neurotransmitter system (Stępnicki *et al*. 2018). While atypicals have demonstrated improved tolerability, they still pose substantial challenges with side effects including weight gain, metabolic disturbances, increased risk of diabetes and cardiovascular complications (Miyamoto *et al*. 2004) and they still lack activity on the cognitive and negative symptoms of the condition (Leucht *et al*. 2013).

In recent years, a growing body of evidence has pointed towards the modulation of glutamatergic neurotransmission as a significant factor in the pathophysiology of schizophrenia (Stone *et al*. 2013), leading to the discovery of a bioactive compound named 3,4,4′-trimethoxychalcone possessing promising neuroprotective activity by acting via the NMDA receptor system using *in silico* models (Chintha & Wudayagiri 2019; Venkataramaiah 2020; Venkataramaiah *et al*. 2021).

Computational techniques have become indispensable tools in preclinical drug design. Today, computer-aided drug design (CADD) is routinely applied in the pharmaceutical industry, yielding substantial results. A recent review reported that between 1981 and 2019, the discovery processes of more than 70 marketed drugs incorporated computational approaches to an extent significant enough to be documented in the scientific literature (Sabe *et al*. 2021). Examples of recently approved drugs are Nirmatrelvir (for SARS-CoV-2 infection) and Sotorasib (for KRAS G12C-mutant lung cancer) which were discovered using CADD methods (Lanman *et al*. 2020; Tuttle *et al*. 2025).

Therefore, this study seeks to design new chalcone derivatives and evaluate their drug-likeness properties and antischizophrenic activity using an in silico method.

## MATERIALS AND METHOD

### Software and Databases

Software and databases used in this research are ChemDraw Ultra 7.0, SwissADME, PubChem Database, ChEMBL, Chemical Database (Chem DB), SureChEMBL, Chemical Entities of Biological Interest (ChEBI), Crystallography Open Database (COD), PubMed, NIST data discovery, ChemSpider, Drug Central, PROTOX 3.0, Spartan14 version 114, Novoprolabs, MarvinSketch version 19.4, MarvinView version 19.4, Chimera 1.11.2, AutoDock Vina, PrankWeb and Discovery Studio version 20.1.

### Protein Crystal Structure

The three-dimensional crystal structure of Human Dopamine D2 receptor with PDB ID 6CM4 in complex with the ligand dopamine and Human Glutamate N2D receptor with PDB ID 30EL were retrieved from the Protein Data Bank (http://www.rscb.org/pdb).

### Design of Chalcone Derivatives

Using the similarity principle as Klein et al. 2002, 10 new chalcone derivatives were designed from 3,4,4′-trimethoxychalcone using ChemDraw Ultra 7.0. Clozapine was used as the reference compound for this study, and its structure was downloaded from the PubChem website (https://pubchem.ncbi.nlm.nih.gov/compound/clozapine).

### Evaluation of Drug Likeness Property and Toxicity Prediction

Predictions of physicochemical, pharmacokinetic and oral bioavailability were made using SwissAdme (Diana *et al*. 2017). Any compound that fails Lipinski’s rule of 5 (Lipinski *et al*. 2001) was excluded from the study. The LD_50_ and toxicity class of all designed compounds were predicted using Protox 3.0 (Drwal *et al*. 2014).

### Protein Preparation

The 3D crystal structures of the proteins intended for the study (rec = GLUN2D receptor PDB ID = 3OEL and rec1 = dopamine D2 receptor PDB ID = 6CM4) were downloaded from Protein Data Bank (http://www.rcsb.org) and purified by removal of residues and native ligands using Chimera version 1.11.2. The files were saved in Protein Data Bank (.pdb) format.

### Ligand Preparation

All designed compounds were converted to 3D using the NovoProLabs (https://www.novoprolabs.com/tools/smiles2pdb) and clozapine which was used as the standard were then optimised in 3D format using Spartan14 version 114 using equilibrium geometry at ground state with semi-empirical calculations PM6. Their molecule (.mol2) file formats were generated and saved for ligand preparation for docking studies. The ligands were further prepared by adding Gasteiger charges using Chimera version 1.11.2. All prepared ligands were saved in .mol2 formats in preparation for docking studies using AutoDock Vina via Chimera.

### Molecular Docking Studies

#### Docking validation

The co-crystalised ligand for both receptors (glutamate for 3OEL and risperidone for 6CM4) were removed using Chimera version 1.11.2 and redocked on the receptors again to validate the method using Autodock Vina via Chimera.

#### Docking of design compounds

Docking was carried out using AutoDock Vina via Chimera 1.11.2. The binding site coordinates for 3OEL as mentioned by Venkataramaiah *et al*. (2021), the centre X: 28.27, Y: 27.13, Z: 28.51, with grid box dimensions 25, 20 and 20 were used to achieve the highest binding scores for the native ligand. The binding site coordinate for 6CM4 was predicted using PrankWeb (http://www.prankweb.cz) with a high probability score (88%) of being X: 9.3303, Y: 6.1417, Z: −10.1226, with grid box dimension of 40, 40, 40, were used. All bound complexes were saved in .pdb formats for further analysis and visualisation.

## RESULTS AND DISCUSSION

### Design

The 10 compounds designed were in their trans-form conforming with the most stable form of chalcones thermodynamically (Zhuang *et al*. 2017), as shown in Table 1.

### Drug Likeness and Oral Bioavailability of the Designed Compounds

All designed compounds exhibited excellent pharmacokinetic properties, including high GI absorption and most having good Blood-Brain barrier permeability. Moreover, the designed compounds possess a better predicted synthetic accessibility score (less than 2.80) than the reference standard, clozapine (3.60), with the synthetic accessibility scores ranging from 1 (very easy) to 10 (very difficult). Additionally, all the designed compounds passed the Lipinski’s rule of 5, with none having any violations. A summary of the predicted pharmacokinetic profiles, lead-likeness properties and bioavailability profiles are shown in Tables 2 and 3.

### Toxicity Profile of the Designed Compound

The designed compounds were found to be relatively safer compared to Clozapine. Their toxicity class range was between 4 and 5 (with 1 being most toxic and 6 being most safe), having LD_50_ values ranging from 500 mg/kg to 2,652 mg/kg as against clozapine that had a predicted class of 3 with 150 mg/kg as its predicted LD_50_ value. In general, from all the parameters in Table 4, it has been shown that some of these designed compounds possess superior toxicity profiles than the reference standard, clozapine.

### Molecular Docking Studies

#### Docking validation

**FIGURE 1:**
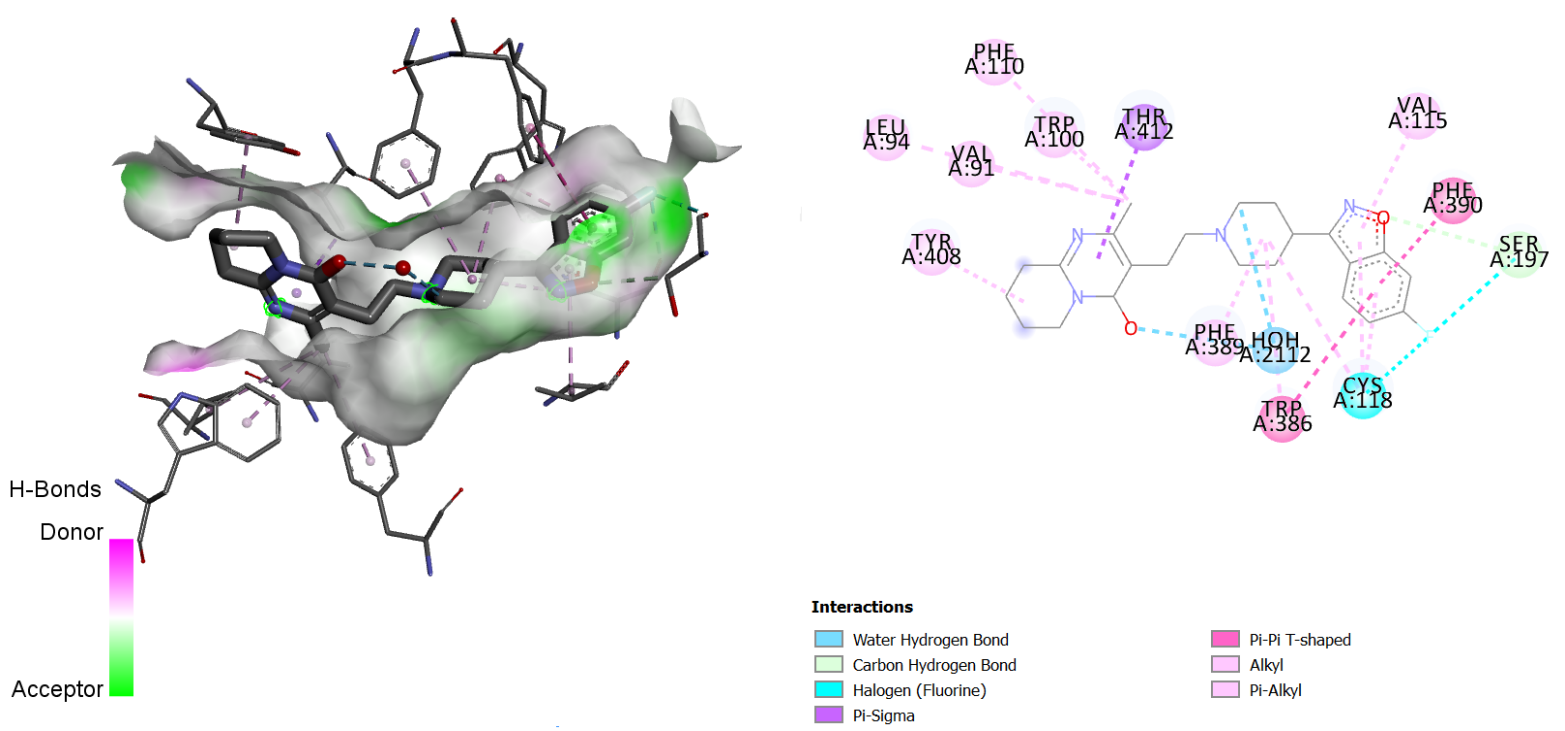
3D/2D structure of co-crystallised native ligand (risperidone) and its interaction with the human Dopamine D2 receptor.

**FIGURE 2:**
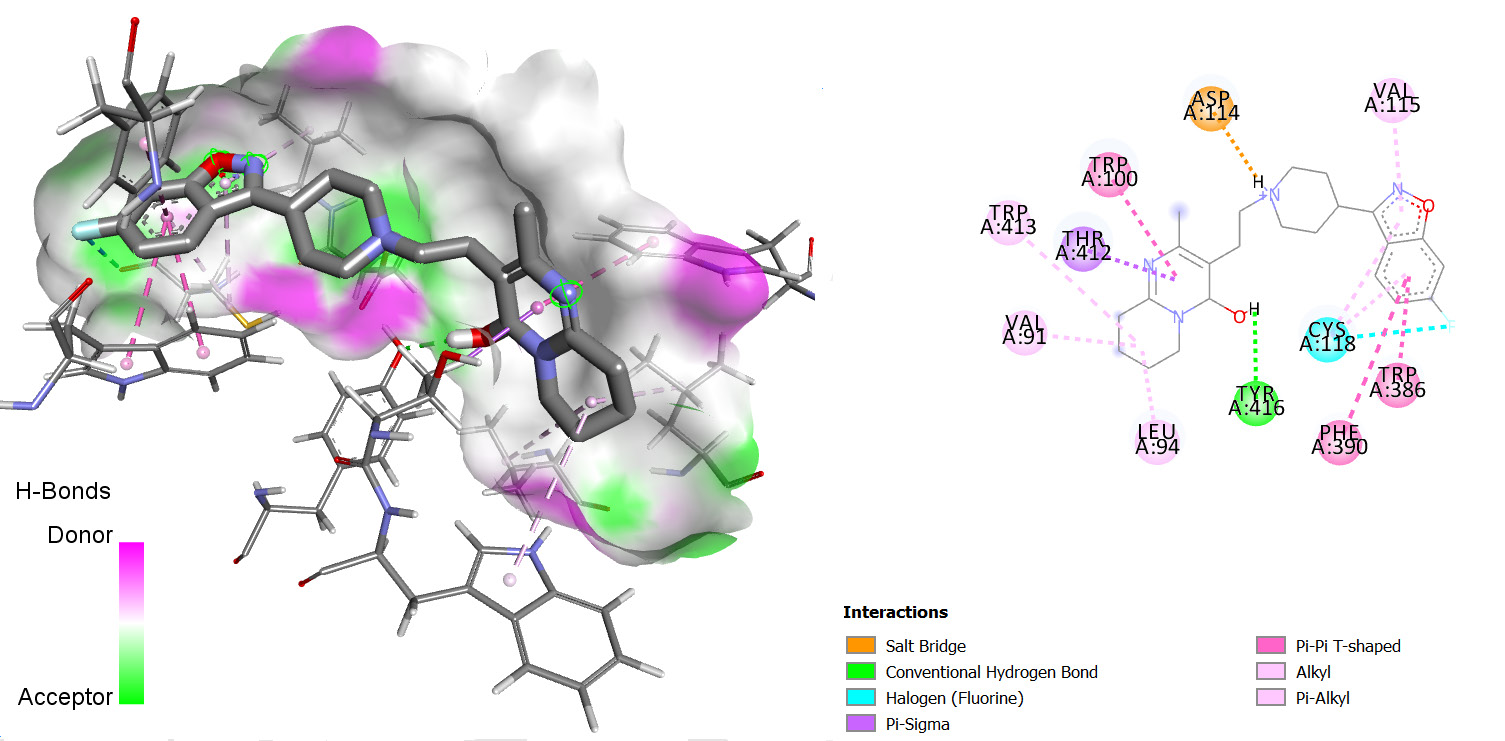
3D/2D structure of co-crystallised native ligand (risperidone) and its interaction with the human Dopamine D2 receptor.

**FIGURE 3:**
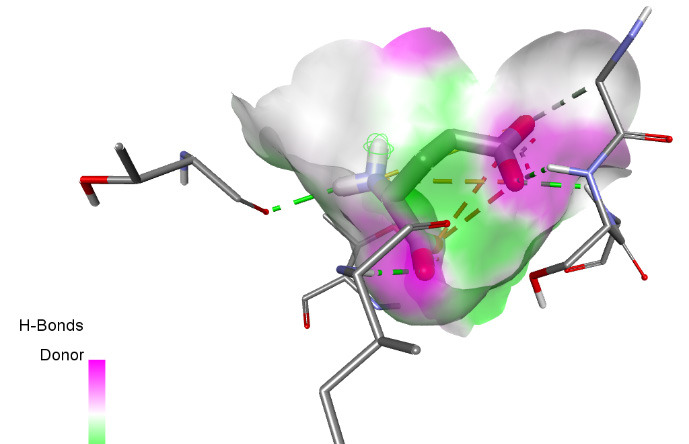

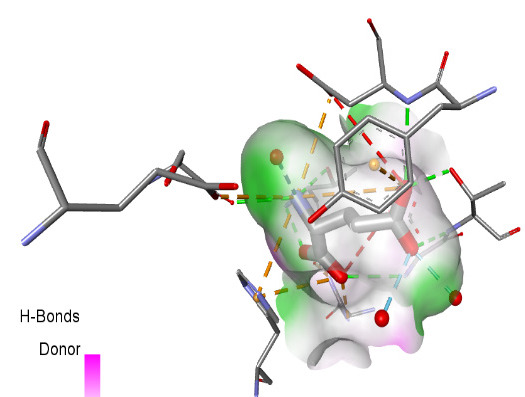
3D/2D image of C6 and its interactions with the Dopamine D2 receptor.

Comparing the redocked native ligand-receptor complex structure and that of the co-crystallised native ligand-receptor complex, they were observed to be very similar, and the native ligand bound to its binding pocket, thus, indicating that we got the pocket co-ordinate and size correctly. The native ligands were observed to interact with the receptors via similar amino acids as shown in Figs. 1–3.

##### Docking studies of chalcone derivatives

The designed chalcone derivatives, C1 to C10 have all shown promising docking ability to Dopamine D2 receptor, with docking scores ranging from −8.3 to −9.5 and number of hydrogen bonds, ranging from 0 to 1, thus illustrating a non-strong bond formation between these compounds and the receptor. A summary of the docking scores and number of hydrogen bonds is shown in Table 5.

**FIGURE 4:**
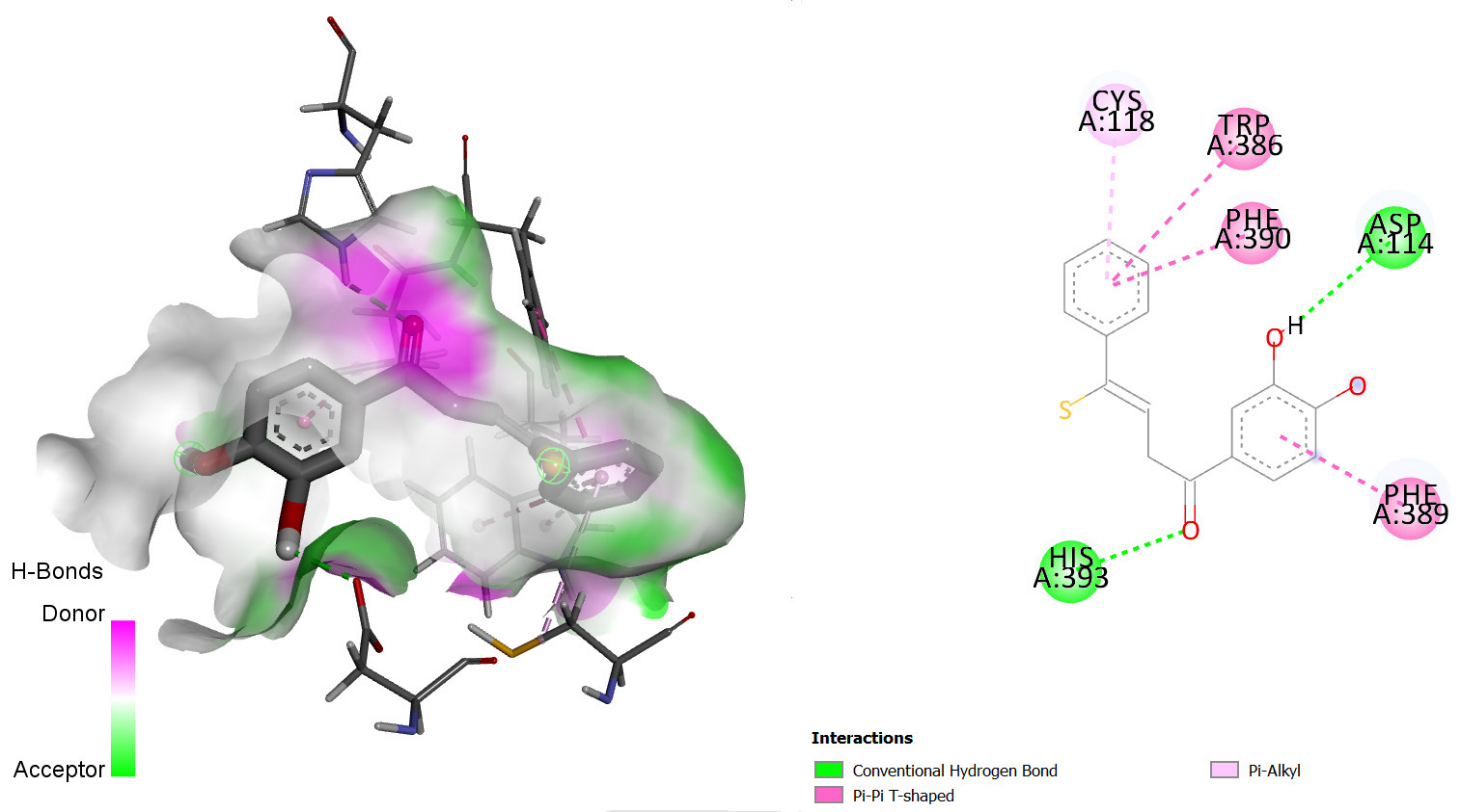
3D/2D image of C6 and its interactions with the Dopamine D2 receptor.

**FIGURE 5:**
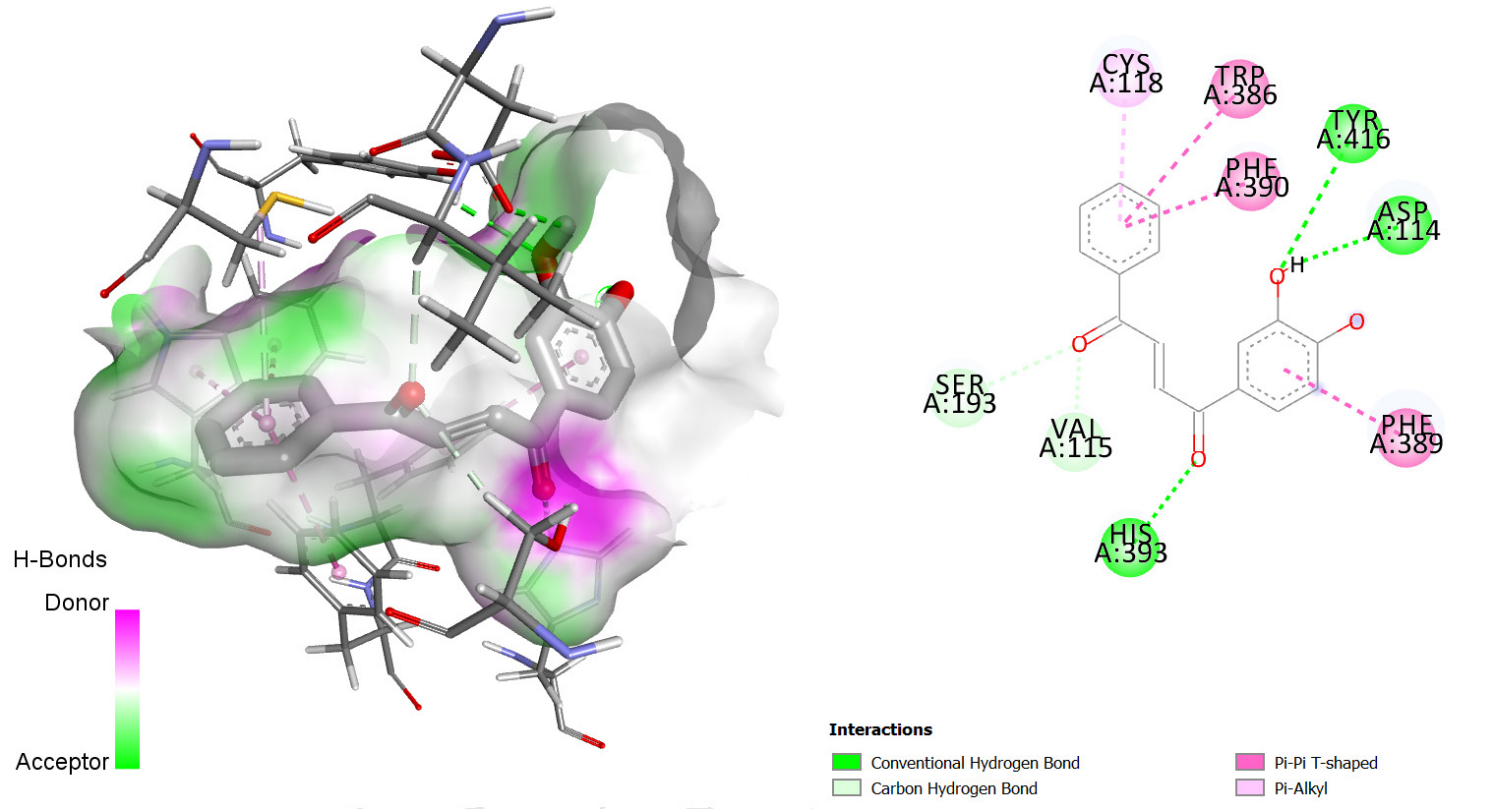
3D/2D image of and its interactions with the Dopamine D2 receptor.

**FIGURE 6:**
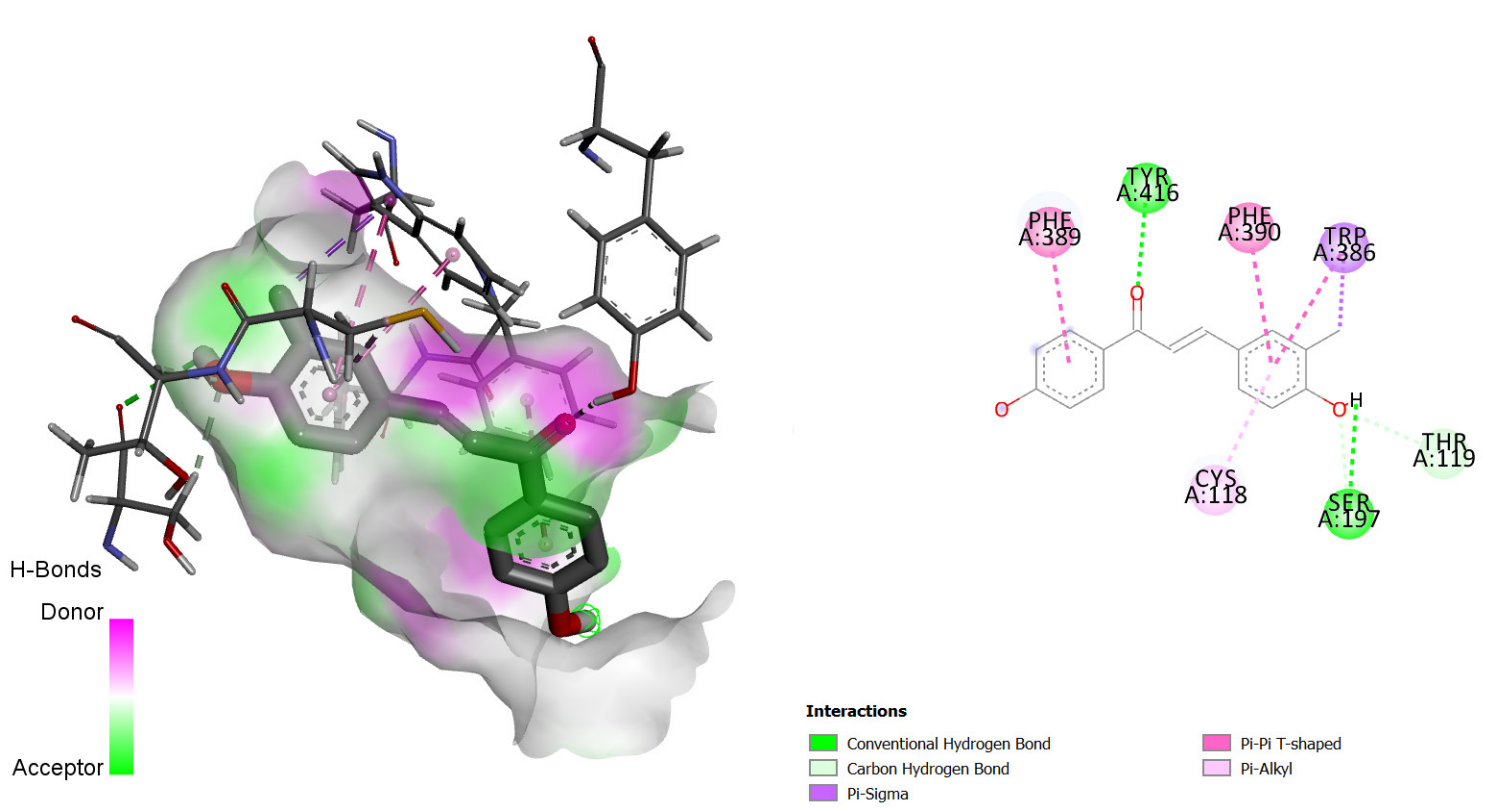
3D/2D image of C8 and its interactions with the Dopamine D2 receptor.

The interactions between some notable ligands (C6, C7 and C8) and Dopamine D2 complex are presented in Figs. 4 to 6.

The designed compounds were docked against Glutamatergic N2D receptor subtype, and all compounds had binding scores very close to the reference standard (clozapine), with their binding scores ranging from −6.0 to −6.6 while that of clozapine was −6.7 (Table 6).

**FIGURE 7:**
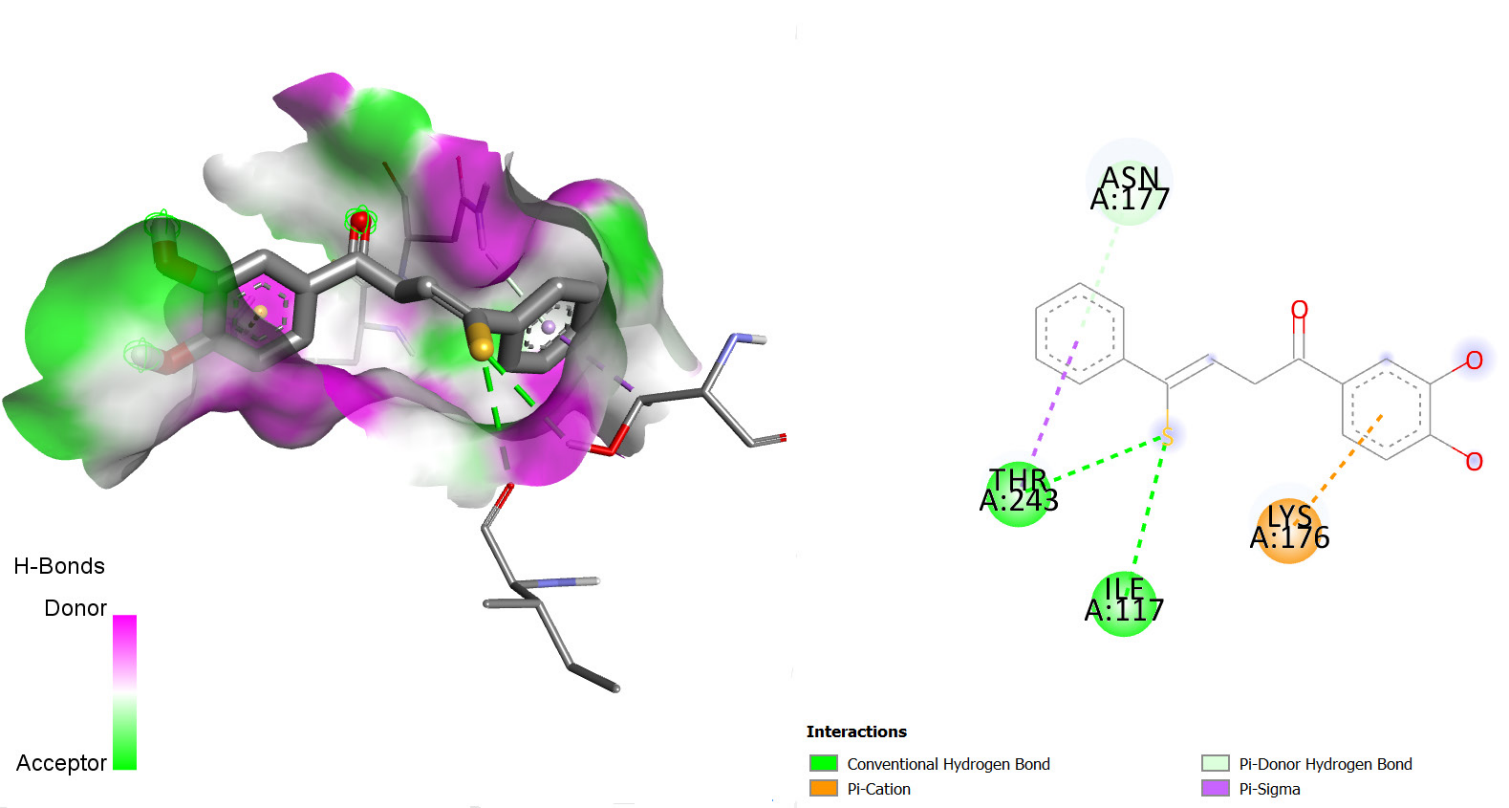
3D/2D image of and its interactions with the Dopamine D2 receptor.

**FIGURE 8:**
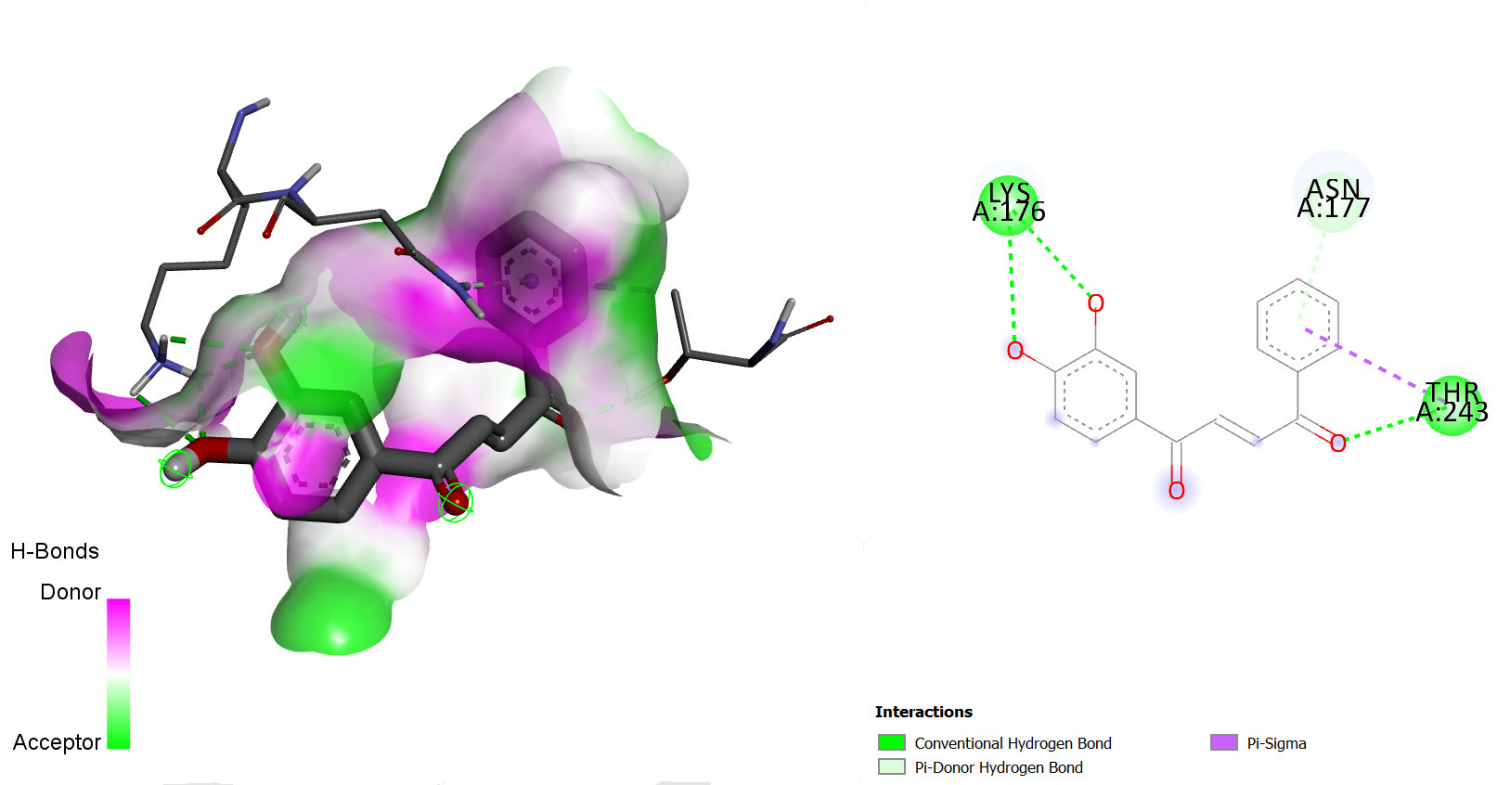
3D/2D image of C7 and its interactions with the glutamate N2D receptor.

**FIGURE 9:**
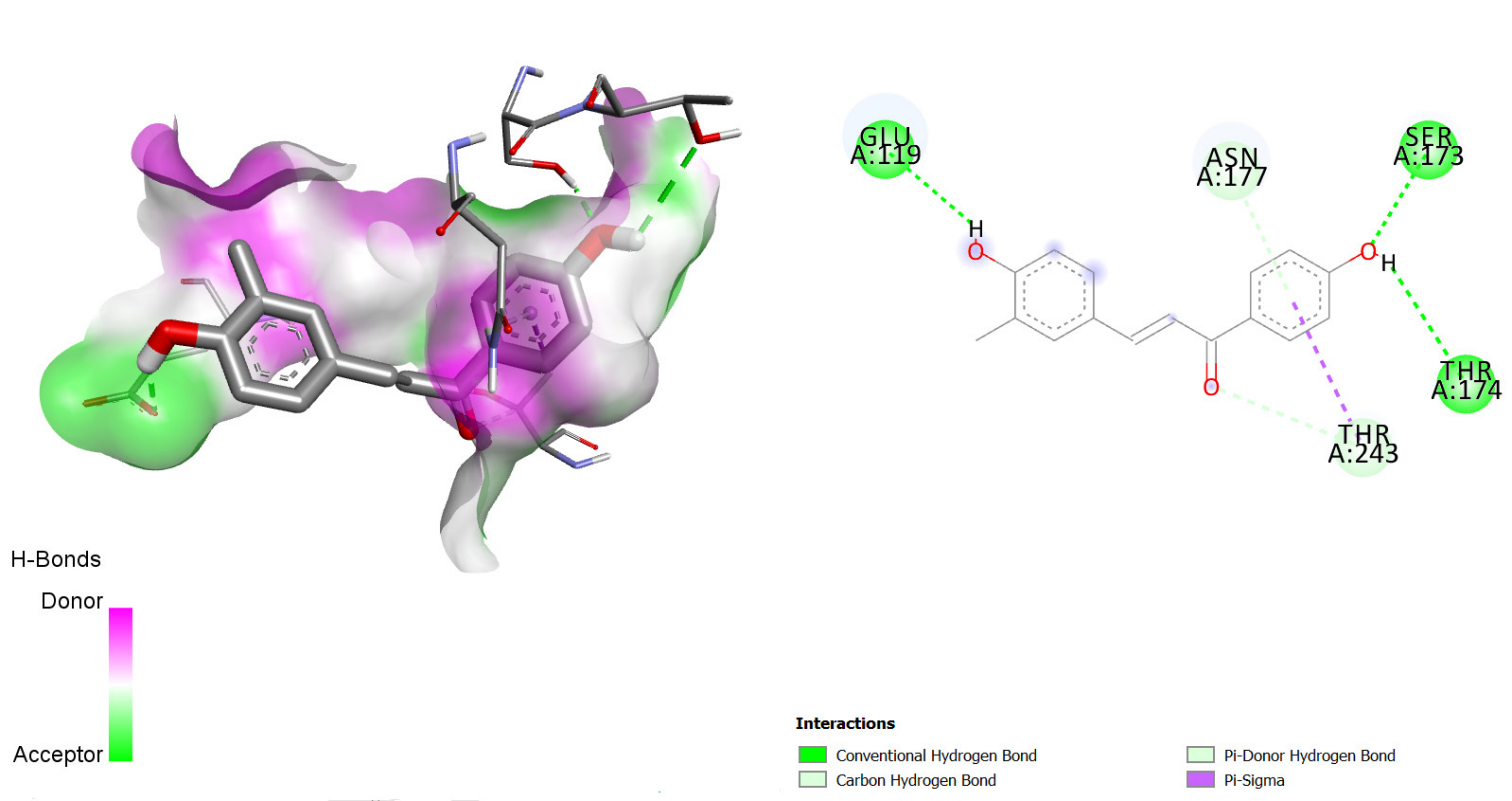
3D/2D image of C8 and its interactions with the glutamate N2D receptor.

The interactions between some notable ligands (C6, C7 and C8) and glutamate NMDA N2D receptor complex are presented in Figs. 7 to 9.

## DISCUSSION

Many promising antischizophrenic therapeutic moieties could not reach phase 1 clinical trials due to unsatisfactory pharmacokinetic disposition relating to absorption, distribution, metabolism, excretion and toxicity (ADMET). These steps are thus critical in determining a drug’s safety and efficacy. The notion of drug-likeness, which seeks to enhance ADMET processes in the human body, has gained considerable interest (Pettersen et al. 2004). To determine oral bioavailability, we examined whether synthesised compounds followed Lipinski’s “Rule of Five”. This rule empirically implies that drugs will have poor oral bioavailability if a molecule has more than five hydrogen bond donors, ten hydrogen bond acceptors, a molecular weight larger than 500 daltons, or a computed Log P value greater than 5. Compounds that violate more than one of these characteristics may struggle with bioavailability and are less likely to have drug-like effects (Zhao *et al*. 2002). Furthermore, the topological polar surface area (TPSA), an important parameter in predicting bioavailability was determined. Typically, compounds with a TPSA larger than 140 angstrom squared (Å^2^) have inadequate bioavailability (Pajouhesh & Lenz 2005) while those having TPSA below 90 have good CNS penetration ability (Hitchcock & Pennington 2006). All designed compounds had TPSA values below 90 Å^2^ except for C10 as shown in Table 2, indicating the CNS penetration ability and high bioavailability.

From the molecular docking studies results, the designed compounds; C6, C7 and C8 showed higher binding affinity to Dopamine D2 receptor with docking scores of 8.9, −9.5 and 9.2 and 1, 1 and 0 hydrogen bonds, respectively. Clozapine on the other hand, as the reference standard, showed a docking score of −8.8 with 1 hydrogen bond. These hydrogen bonds have been reported to strengthen the stability of the protein-ligand complex formed (Fu *et al*. 2018).

Compound C6 had 3 pi-pi T-shaped bond interactions with TRP A:386, PHE A:390 PHE A:389 of the Dopamine D2 receptor. It also showed a pi-akyl bond interaction with CYS A:118 of the receptor and 2 conventional H-bond interactions with ASP A:114 and HIS A:398.

Compound C7 on the other hand, showed similar bond interactions with C6 with the conventional H-bond interactions in addition to that of C6, an extra one with TYR A:416 and 2 van der Waals bond interactions with SER A:193 and VAL A:115 of the receptor. These van der Waals interactions are also very important just as the hydrogen bond formation, in determining whether a ligand will bind to a protein or not (Menchaca *et al*. 2020).

Compound C8, showed 2 pi-pi alkyl bond interactions with PHE A390 and PHE A:389 of the receptor, a pi-sigma bond interaction with TRP A:386, 2 conventional hydrogen bonds with TYR A:416 and SER A:197, a van der Waals bond interaction with THR A:119 and a Carbon-Hydrogen bond interaction with SER A:197. As for Clozapine, the reference standard, it showed 3 pi-Alkyl bond interactions with TRP A:413, VAL A:91 and LEU A:94 one salt bride with ASP A:114 and one unfavourable donor bond with THR A:412.

In addition, in recent years, a growing body of evidence has pointed towards the modulation of glutamatergic neurotransmission as a significant factor in the pathophysiology of schizophrenia (Stone *et al*. 2013), For this research Glutamatergic N2D receptor subtype was selected for binding studies with the designed chalcone derivatives, C1 to C10. As shown in Table 6, all compounds had binding scores very close to the reference standard (clozapine), with their binding scores ranging from −6.0 to −6.6 while that of clozapine was −6.7.

Various types of binding interactions were observed including pi-pi alkyl bond, which contributes to the overall stability of the complex and enhances the binding affinity (Abdullahi & Adeniji 2020), conventional hydrogen bonds, pi-alkyl bonds, pi-sulfur bonds, van der Waals bonds amidst many others as seen in Figs. 7 to 9.

While *in silico* modelling provides powerful insights into molecular interactions and accelerates the identification of potential drug candidates, experimental validation remains indispensable in confirming the biological relevance of these predictions (Niazi & Mariam 2023). Computational approaches such as molecular docking, and Molecular Dynamics (MD) simulations can reliably propose binding modes, affinities and structure–activity relationships; however, these predictions are inherently dependent on the quality of input data, scoring functions, and underlying algorithms, which can introduce biases or false positives. Therefore, rigorous in vitro and in vivo validation is essential to verify predicted activity, assess pharmacokinetics, toxicity and off-target effects, and ultimately establish clinical relevance (Mellini *et al*. 2019; Ouma *et al*. 2024; Ugbaja *et al*. 2025).

## CONCLUSION

Ten new compounds were designed by modifying the structures of various chalcones and they were found to have high binding affinity to the human dopamine receptor and a relatively high binding affinity to glutamate N2D NMDA receptor, suggesting that they have some antischizophrenic activity. Their oral bioavailability was predicted to be excellent and toxicity profiles were predicted to be very safe relative to the reference standard, making them potential drug candidates. Although the new 1,3-disubstituted chalcone derivatives demonstrated high binding affinity to the human dopamine receptor and glutamate N2D NMDA receptor using in silico molecular docking study, further studies on the molecular dynamic simulation (Root Mean Square Dimension, Root Mean Square Fluctuation, Radius of Gyration, Solvent Accessible Surface Area) should be carried out to fully understand and validate the binding and stability of the protein-ligand complex.

## Figures and Tables

**TABLE 1:**
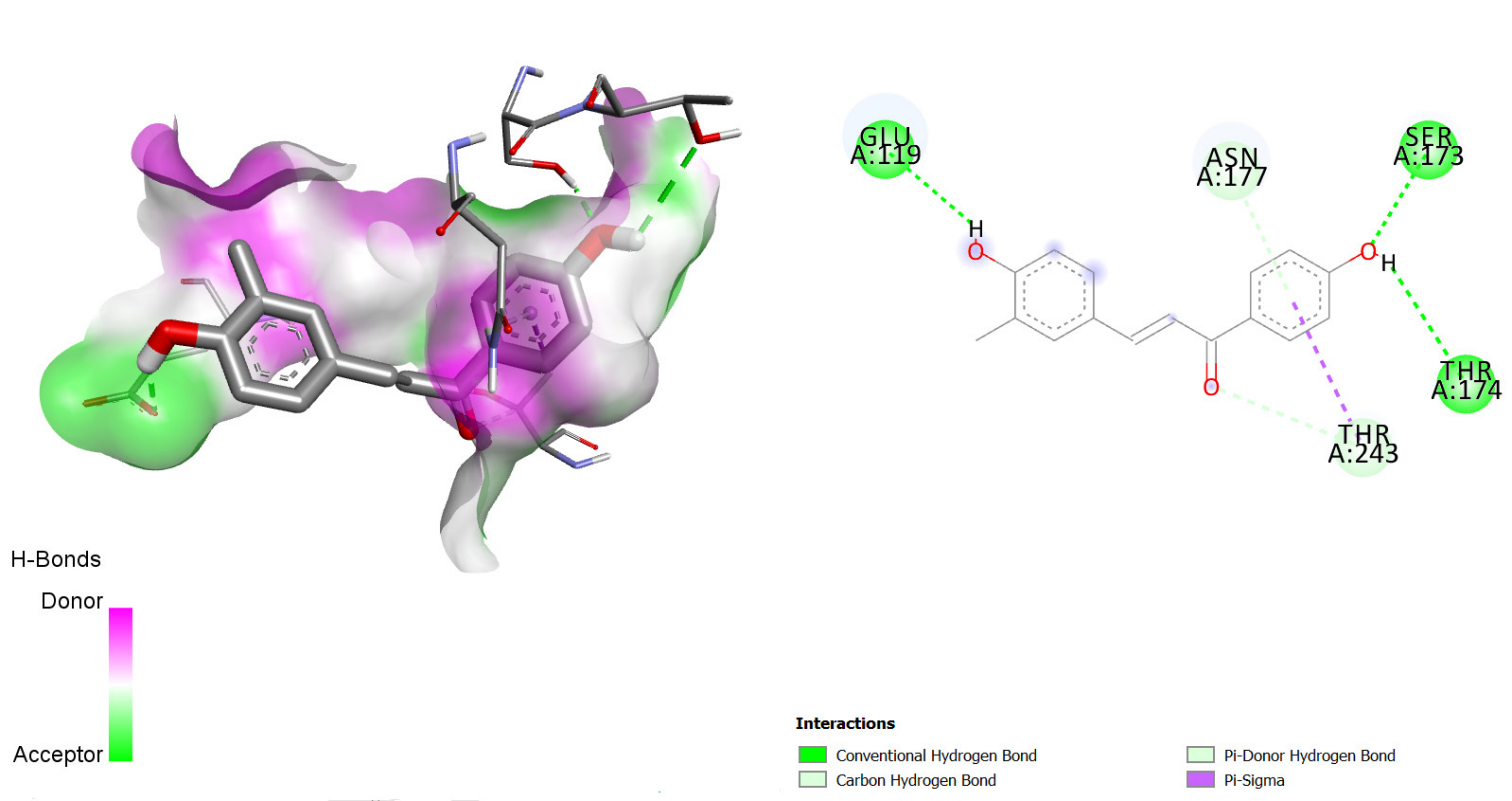
Canonical smiles and 2-dimensional structures of designed chalcone derivatives.

**TABLE 2:** Predicted drug-likeness properties of designed chalcone derivatives and reference (Clozapine) analysed with SwissADME.

Compounds	Molecular weight (g/mol)	LogP	nHBD	nHBA	TPSA	MR	Log k_p_ (cm/s)	Log S	nRotB	Lipinski’s violation	Remark
C1	300.31	1.52	5	3	86.99	83.62	−1.94	−6.03	5	0	PASS
C2	286.35	3.68	3	1	85.33	82.02	−3.26	−5.59	4	0	PASS
C3	298.33	2.31	4	2	66.76	86.40	−2.09	−5.39	6	0	PASS
C4	284.31	2.08	2	4	66.76	81.59	−2.20	−5.08	5	0	PASS
C5	284.31	2.08	2	4	66.76	81.59	−2.74	−5.68	5	0	PASS
C6	284.33	2.34	2	3	89.62	82.10	−7.52	−5.85	4	0	PASS
C7	268.26	1.49	2	4	74.60	74.73	−7.48	−6.18	4	0	PASS
C8	254.28	2.42	2	3	57.23	75.26	−7.50	−5.48	3	0	PASS
C9	286.35	2.68	1	3	85.33	82.02	−7.04	−5.59	4	0	PASS
C10	272.32	2.44	2	3	96.33	77.55	−3.66	−5.74	3	0	PASS
Clozapine	326.82	3.31	1	2	30.87	110.05	−3.88	−6.00	1	0	PASS

*Notes:* Linpinski’s rule of 5: (1) MW < 500Da; (2) LogP < 5; (3) nHBD < 5; (4) nHBA < 10; (5) nRotB < 5.

Abbreviations: MW = Molecular weight, LogP = Log of octanol/water partition coefficient, nHBA = Number of hydrogen bond acceptor(s), nHBD = Number of hydrogen bond donor(s), MR-Molar

**TABLE 3:** Predicted bioavailability properties of designed chalcone derivatives and reference Clozapine analysed with SwissADME.

Compounds	Log-K_p_ (cm/s)	GI abs	BB perm	P-gp substrate	CYP1A2 inhibitor	CYP2C19 inhibitor	CYP2C9 inhibitor	CYP2D6 inhibitor	CYP3A4 inhibitor
C1	−1.94	HIGH	NO	NO	YES	NO	YES	NO	YES
C2	−3.26	HIGH	NO	NO	YES	YES	YES	NO	YES
C3	−2.09	HIGH	YES	NO	YES	YES	YES	YES	YES
C4	−2.20	HIGH	YES	NO	YES	YES	YES	NO	YES
C5	−2.74	HIGH	YES	NO	YES	YES	YES	NO	YES
C6	−7.52	HIGH	NO	NO	YES	NO	YES	NO	YES
C7	−7.48	HIGH	YES	NO	YES	NO	NO	NO	YES
C8	−7.50	HIGH	YES	NO	YES	NO	NO	NO	YES
C9	−7.04	HIGH	NO	NO	YES	YES	YES	NO	YES
C10	−3.66	HIGH	NO	NO	YES	NO	YES	NO	YES
Clozapine	−3.88	HIGH	YES	YES	YES	NO	NO	YES	YES

*Notes:* log K_p_ = Log of skin permeability; GI Abs = Gastro-intestinal absorption; BBB Per = Blood-Brain barrier permeability; P-gp = P-glycoprotein; CYP = cytochrome-P450.

**TABLE 4:** Predicted toxicity properties for designed chalcone derivatives.

Compounds	Predicted LD_50_ (mg/kg)	Predicted toxicity class (1 to 6)	Average similarity (%)	Prediction accuracy (%)
C1	2,652	5	79.75	69.26
C2	2,100	5	66.03	68.07
C3	2,652	5	77.20	69.26
C4	2,652	5	80.59	70.57
C5	2,100	5	83.66	70.97
C6	500	4	63.93	68.07
C7	500	4	75.00	69.26
C8	1,048	4	81.62	70.97
C9	2,652	5	72.07	69.26
C10	1,000	4	68.21	68.07
Clozapine	150	3	100	100

**TABLE 5:** Docking scores and number of hydrogen bonds for designed chalcone derivatives in complex with Dopamine D2 receptor.

Compounds	Binding score	No. of hydrogen bonds
Clozapine	−8.8	1
C1	−8.6	0
C2	−8.3	0
C3	−8.7	0
C4	−8.7	0
C5	−8.7	1
C6	−8.9	1
C7	−9.5	1
C8	−9.2	0
C9	−8.3	1
C10	−8.5	1

**TABLE 6:** Docking scores and no. of hydrogen bonds for designed chalcone derivatives in complex with GluN2D NMDA receptor.

Compounds	Binding score	No. of hydrogen bonds
Clozapine	−6.7	0
C1	−6.6	1
C2	−6.0	1
C3	−6.6	2
C4	−6.4	2
C5	−6.5	2
C6	−6.6	1
C7	−6.5	1
C8	−6.5	1
C9	−6.1	0
C10	−6.3	1
